# Endometrial Adenocarcinoma and Mucocele of the Appendix: An Unusual Coexistence

**DOI:** 10.1155/2013/892378

**Published:** 2013-05-16

**Authors:** Ioannis Kalogiannidis, Amalia Mavrona, Sophia Grammenou, Georgios Zacharioudakis, Stamatia Aggelidou, David Rousso

**Affiliations:** ^1^3rd Department of Obstetrics and Gynecology, Aristotle University of Thessaloniki, Konstantinoupoleos 49, 54624 Thessaloniki, Greece; ^2^5th Department of General Surgery, Aristotle University of Thessaloniki, Konstantinoupoleos 49, 54624 Thessaloniki, Greece; ^3^Department of Pathology, Hippokration General Hospital, Konstantinoupoleos 49, 54624 Thessaloniki, Greece

## Abstract

Appendiceal mucocele is a rare clinical entity, which is however quite often associated with mucinous ovarian tumor. The coexistence of mucinous cystadenoma of the appendix and endometrial adenocarcinoma has not been reported before. A 49-year-old woman presented to our clinic with postmenopausal bleeding and no other symptom. Endometrial biopsy revealed endometrial adenocarcinoma of endometrioid type (grade I). Preoperative CT scanning revealed an appendiceal mucocele, and a colonoscopy confirmed the diagnosis. The patient underwent total abdominal hysterectomy with bilateral salpingo-oophorectomy and appendectomy. The final histopathological examination showed a mucinous cystadenoma of the appendix and confirmed the diagnosis of endometrioid endometrial adenocarcinoma. The coexistence of appendiceal mucocele and female genital tract pathology is rare. However, gynecologists should keep a high level of suspicion for such possible coexistence. Both the diagnostic approach and the therapeutic management should be multidisciplinary, most importantly with the involvement of general surgeons.

## 1. Introduction

Appendiceal mucocele is an uncommon but well-characterized entity that can either present with clinical symptoms or as an incidental radiological or surgical finding. The term includes three histopathological types, that is, mucosal hyperplasia, mucinous cystadenoma, and mucinous cystadenocarcinoma, and the overall prevalence is reported to be 0.2–0.4% among appendectomies. More than half of appendiceal mucoceles are mucinous cystadenomas, have low malignant potential, and can be treated with appendectomy alone, combined with thorough exploratory laparotomy for mucinous peritoneal adhesions (pseudomyxoma peritonei). Notwithstanding, appendiceal mucinous tumors have repeatedly been reported to coexist with ovarian epithelial tumors, especially of mucinous type [1–3]. In fact, some experts recommend routine appendectomy during ovarian cancer surgery, mainly to rule out the presence of microscopic metastases or the possibility of a primary appendiceal adenocarcinoma. However, the coexistence of appendiceal mucocele and endometrial adenocarcinoma has not been reported before. 

We present a case of a postmenopausal woman with endometrial adenocarcinoma that was diagnosed with appendiceal mucocele during the preoperative radiological staging with computed tomography (CT) scan, in the context of the relevant literature.

## 2. Case Report

A 49-year-old Caucasian woman with two previous pregnancies (gravida 2) presented to our outpatient department with postmenopausal bleeding and no other symptoms. The patient's medical history included a pituitary microadenoma that was operated a year ago, ACTH-dependent Cushing, type II diabetes mellitus, and central type hypothyroidism. 

Transvaginal ultrasonography revealed multilocular cystic masses in both ovaries, while the endometrial thickness of the uterus was 14 mm. To further investigate the ovarian cystic masses, CT scan of the abdomen was performed. The CT scan confirmed the ultrasonographic findings regarding the ovaries and revealed an appendiceal mucocele with mural calcification. All the serum tumor markers (CA 125, CA 19–9, CEA) were normal. The patient was subjected to colonoscopy according to the recommendation of the surgical team that was involved in our diagnostic workup, which confirmed the diagnosis of appendiceal mucocele. Endometrial biopsy by dilatation and curettage (D&C) revealed endometrial adenocarcinoma of endometrioid type (type I), well differentiated (grade 1).

**Figure 1:**
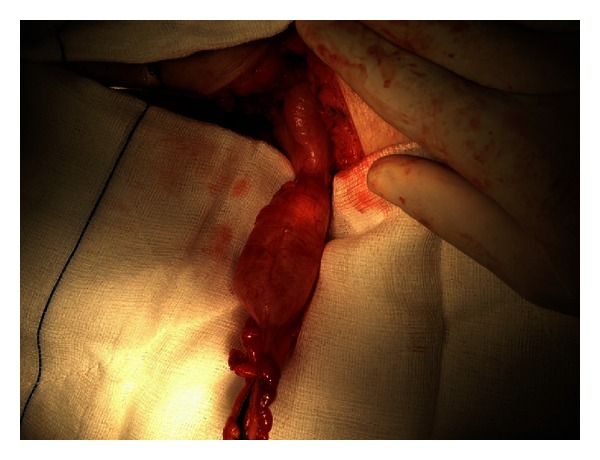
Intraoperative view of mucocele of the appendix.

**Figure 2:**
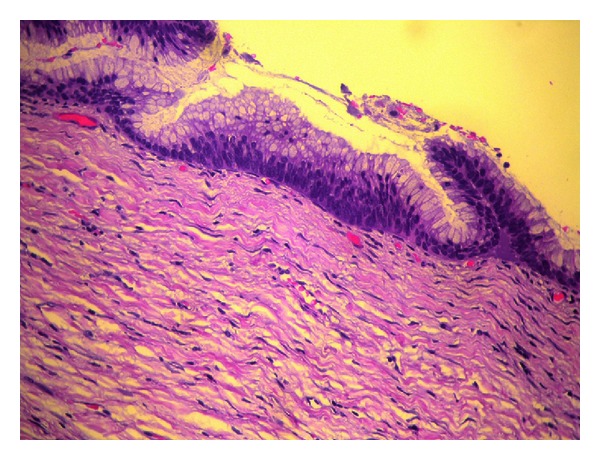
Mucinous cystadenoma of the appendix. Hematoxylin eosin stain.

**Figure 3:**
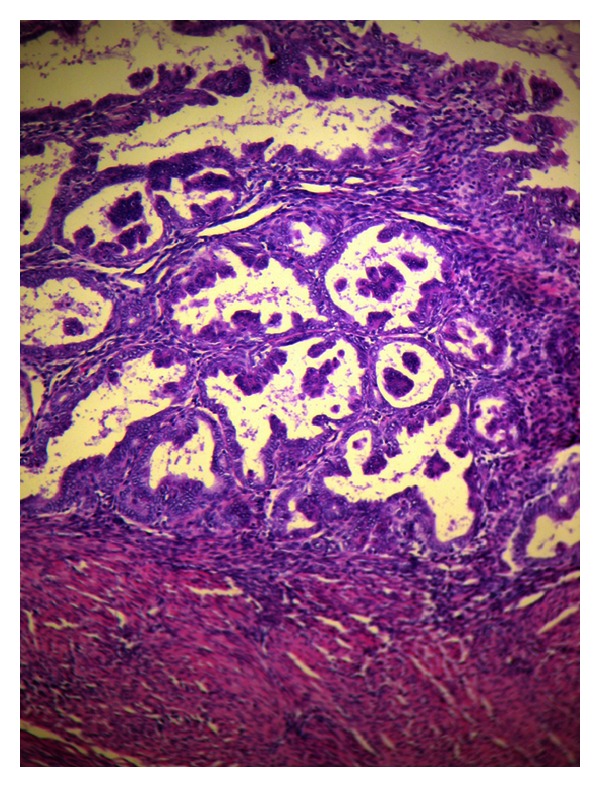
Endometrioid adenocarcinoma FIGO Ia (grade 1). Hematoxylin eosin stain.

The patient underwent total abdominal hysterectomy with bilateral salpingo-oophorectomy as well as intact appendectomy (Figure 1) with the use of staplers performed by a general surgeon. The final histopathological examination sets the diagnosis of mucinous appendiceal cystadenoma (Figure 2), endometrial adenocarcinoma (type I, grade I), (Figure 3), and benign cysts of both ovaries.

## 3. Discussion

An appendiceal mucocele was first described by Rokitansky in 1842 and characterized in 1973 by Aho et al., [4] with the term retention cyst to describe a sterile outflow obstruction in the appendix that was dilated and swollen with glary mucus. There are three histopathological types of mucoceles: (a) mucosal hyperplasia (5%–25% of appendiceal mucoceles), (b) mucinous cystadenoma (as in the present case) which represents 63%–84% of mucoceles and is characterized by low-grade epithelial dysplasia, and (c) mucinous cystadenocarcinoma (11%–20% of mucoceles) [4]. These types are more likely to have a diameter greater than 2 cm. Mucoceles of less than 2 cm in diameter are usually simple retention cysts [4, 5].

In 50% of cases, a palpable mass is found and the patient complains of abdominal pain in the right lower quadrant. In other cases, apparent cystic structure may be an incidental finding during a routine gynecologic screening. The ultrasound of the abdomen often mimics an adnexal mass. CT scan is the ideal imaging method for the diagnosis of appendiceal mucoceles [1, 6].

Both mucinous cystadenoma and cystadenocarcinoma have the potential to seed the peritoneum, leading to pseudomyxoma peritonei or mucinous carcinomatosis. Consequently, removal of the appendix requires caution to avoid potential rupture of the mucocele [1, 7].

The best surgical management of such patients remains controversial. There is no consensus regarding either the optimal surgical procedure, which may be right hemicolectomy or simple appendectomy, or the optimal surgical technique, that is, laparoscopy versus laparotomy [8]. While the right hemicolectomy was associated with a survival advantage concerning the treatment of mucinous cystadenocarcinoma, most recent prospective data demonstrate no benefit in terms of potential pseudomyxoma peritonei or mucinous carcinomatosis in this group of patients [8–10]. Nevertheless, precautions should be taken in order to prevent dissemination of mucus, such as gentle handling of the appendix. In the absence of local invasion, appendectomy with mesoappendix excision appears to be the optimal treatment approach. However, in case of cecal or colon involvement right hemicolectomy is obligatory [8]. Regarding the surgical technique, some authors claim that laparotomy allows better visualization of the abdominal cavity and identification of potential pseudomyxoma as compared to the laparoscopic approach [11]. Conversely, laparoscopy has been associated with a higher incidence of peritoneal implants and inadvertently missed lesions [8, 11].

Mucinous tumors of the appendix and ovary can occur simultaneously in association with pseudomyxoma peritonei. The pathogenetic relationship between the two side tumors, as well as the primary origin, remains obscure. It has been supported that the origin of such mucinous tumors comes from gastrointestinal tract and more specifically from the appendix [1, 7]. There is also a theory that the mucinous ovarian tumors are the result of spread from the appendix to the ovary via the peritoneum. On the other hand, some suggest that these tumors are independent primary neoplasms that develop as a result of a neoplastic field change that affects colonic type epithelium [2, 12]. According to the previous theories, appendectomy is recommended when frozen section diagnosis is mucinous ovarian tumor independently of the benign, borderline, or malignant nature of the neoplasm, in order to eliminate the gastrointestinal origin of the tumor [3].

Apart from the coexistence of appendiceal mucocele and ovarian tumors, appendiceal pathology may also be present in patients with cystic fibrosis or carcinoid, although being rare [13]. Concerning the female genital tract, endometriosis may also coexist with appendicular pathology. Thus, it appears that appendiceal mucoceles relate to heterogeneous surgical or pathological manifestations [14].

To the best of our knowledge, this is the first reported case where appendiceal mucocele was found in combination with endometrial adenocarcinoma. It is not clear whether there is a common underlying pathogenetic mechanism for this uncommon coexistence. Gynecologists should be able to integrate the identification of an appendiceal mucocele in their diagnostic approach, focusing on ovarian pathologic structures, but at the same time staying alert for additional pathology of the female genital tract, such as the endometrium. In such cases, the diagnostic and therapeutic approach needs to be multidisciplinary, and general surgeons should be involved so as the optimal approach of the appendicular pathology is ensured.
